# Heterotaxy syndrome – An unusual cause for bowel obstruction in an adult

**DOI:** 10.4102/sajr.v28i1.2831

**Published:** 2024-05-30

**Authors:** Radhiya Minty, Tanaka Gomba, Rabia Abid

**Affiliations:** 1 Department of Radiology, Faculty of Health Sciences, University of the Witwatersrand, Johannesburg, South Africa

**Keywords:** heterotaxy, polysplenia syndrome, left isomerism, situs ambiguous, bowel obstruction

## Abstract

Heterotaxy syndrome, also known as situs ambiguous, is a spectrum of pathology due to loss of the normal right-to-left asymmetry of the thoraco-abdominal organs. This report describes the case of a 39-year-old female, previously well, who presented with bowel obstruction. CT showed features of left isomerism, with malrotation and volvulus.

**Contribution:**

Heterotaxy syndrome consists of cardiac and non-cardiac manifestations. Imaging studies play a crucial role in the individualised management of the patient.

## Introduction

The normal anatomical arrangement of thoracic and abdominal organs is known as situs solitus, where there is an expected asymmetry of the thoraco-abdominal organs. Heterotaxy syndrome is a spectrum of pathology due to loss of the normal left-to-right asymmetry of the thoraco-abdominal organs.^1^ The incidence of heterotaxy syndrome is 1 per 10 000–20 000 live births. It has been associated with various modes of genetic transmission, although the majority of cases are sporadic.^2^

## Case report

A 39-year-old female, with no known co-morbidities, presented to the emergency department with a 1-week history of colicky upper abdominal pain, vomiting, constipation and loss of appetite. On physical examination, she was apyrexial, with normal vital signs; her abdomen was distended, with high-pitched bowel sounds and generalised abdominal tenderness. On the day of admission, her haemoglobin was 9 g/dL, white cell count was 7.11 × 10^9^/L and C-reactive protein was elevated at 161.

The abdominal radiograph showed multiple distended small bowel loops with air-fluid levels, no free air under the diaphragm and no pneumatosis intestinalis. The bones and soft tissues were normal.

**FIGURE 1:**
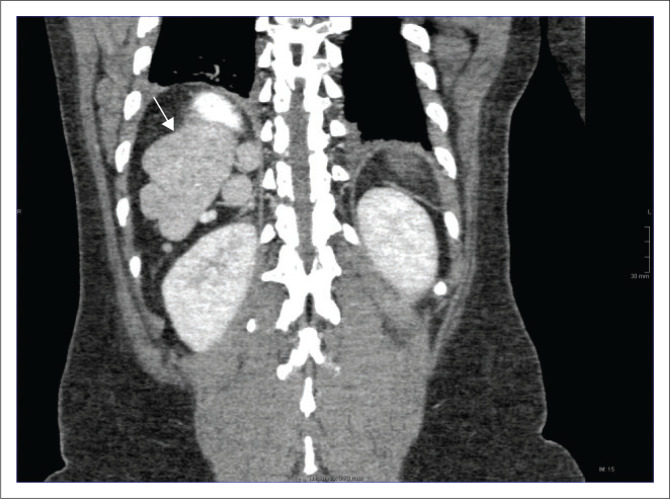
Coronal contrast-enhanced CT image demonstrating multiple right-sided splenules with a parent spleen (white arrow).

**FIGURE 2:**
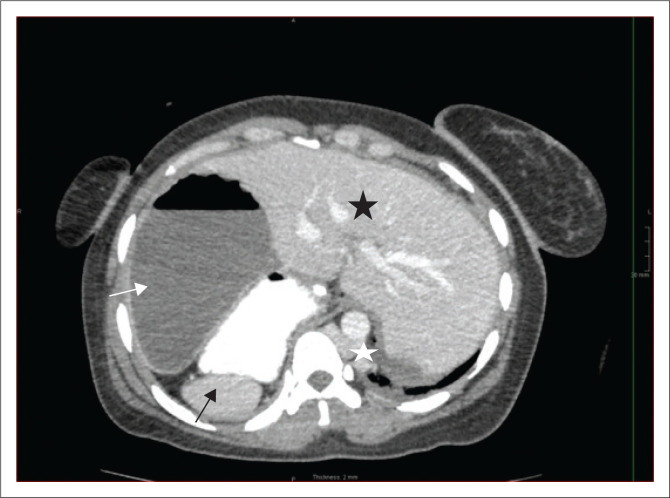
Axial contrast-enhanced CT image demonstrating the liver located on the left and in the midline (black star), a right-sided stomach (white arrow) and a right-sided spleen (black arrow). The IVC is left-sided in the abdomen (white star).

**FIGURE 3:**
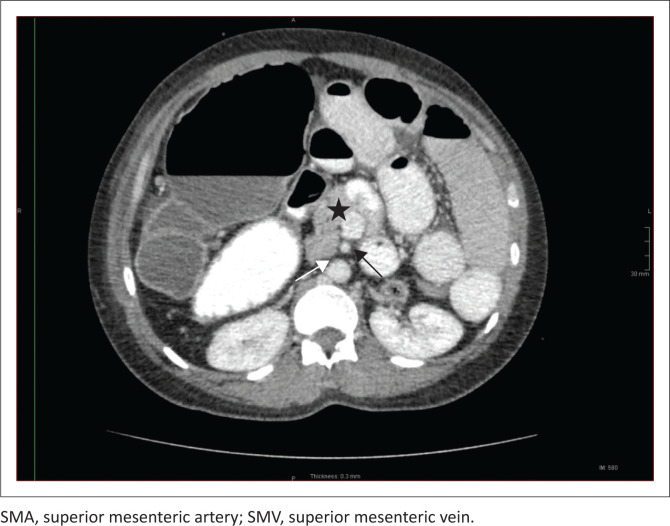
Axial contrast-enhanced CT image demonstrating hypoplasia of the pancreatic body and tail, suggestive of a truncated pancreas (black star), absence of the retroperitoneal course of the duodenum (white arrow) and a parallel vertical relationship of the SMA and SMV (black arrow).

**FIGURE 4:**
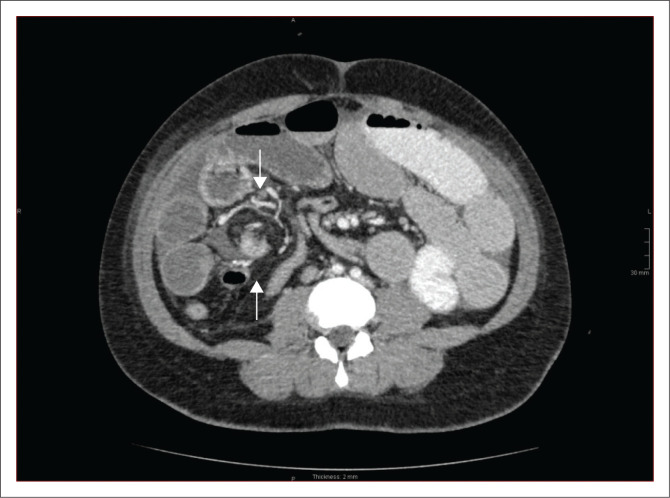
Axial contrast-enhanced CT image showing the ‘whirl sign’, twisting of the mesentery and mesenteric vessels (white arrows).

**FIGURE 5:**
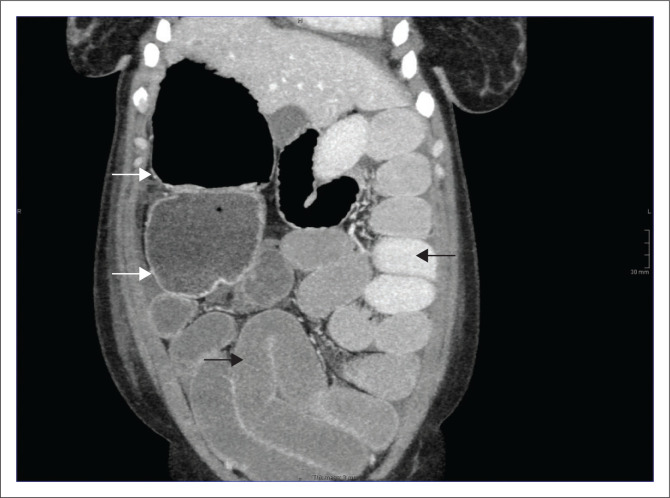
Coronal contrast-enhanced CT image showing multiple dilated loops of small bowel (black arrow) and a massively dilated caecum and proximal ascending colon (white arrows).

A CT of the abdomen revealed right-sided polysplenia (Figure 1) with a dominant parent spleen, a left-sided liver, extending across the midline, and a right-sided stomach (Figure 2). The superior mesenteric artery and vein were parallel to one another (Figure 3). The duodenum coursed anterior to the portosplenic vein with the duodenojejunal junction located to the left of the left-sided vertebral body pedicle but inferior to the level of the duodenal bulb, in keeping with some degree of malrotation. The jejunal loops were located in the left side of the abdomen. There was twisting of the mesentery in the right lower quadrant (Figure 4) with small bowel dilatation and massive dilatation of the caecum and proximal ascending colon (Figure 5). The transition point was within the proximal to mid ascending colon, with collapse of the large bowel distal to this point. There was also dilatation of the small bowel, most likely due to an incompetent ileocecal valve. A small amount of free fluid was noted in the right paracolic gutter and the Pouch of Douglas with multiple mesenteric lymph nodes in the right iliac fossa. Additionally, there was absence of the uncinate process of the pancreas suggestive of a truncated pancreas (Figure 3).

**FIGURE 6:**
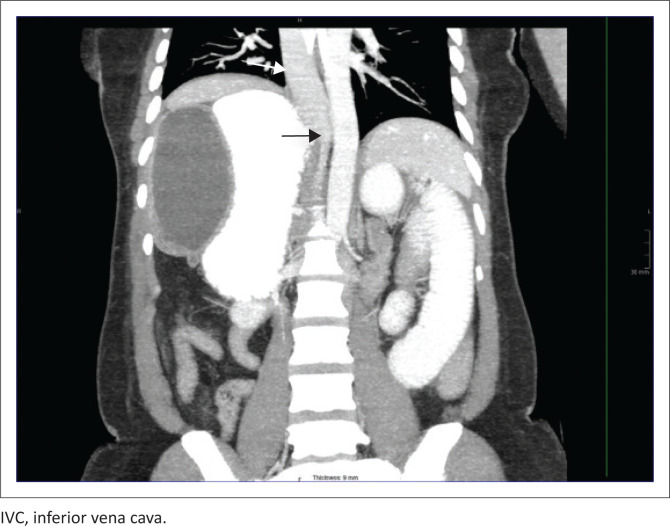
Coronal contrast-enhanced CT image showing the IVC crossing the midline at the level of the diaphragm (black arrow), and azygous continuation of the IVC above the level diaphragm (white arrow).

The chest CT demonstrated a left-sided inferior vena cava (IVC) within the abdomen. Above the diaphragm, the IVC crossed the midline and continued into the chest as the azygous vein. There was no hepatic IVC (Figure 6). There was a common origin of the left common carotid artery and brachiocephalic trunk, in keeping with a Bovine aortic arch. The right lung was trilobed with an eparterial bronchus. The left lung was bilobed with a hyparterial bronchus.

The electrocardiogram was normal with a normal rate, rhythm and axis. Echocardiography revealed a structurally normal heart with normal cardiac function.

The patient proceeded to theatre for an urgent exploratory laparotomy and was found to have simple ascites, a short, small bowel mesenteric root, Ladd’s bands along the duodenum and midgut volvulus. The bowel was viable with no features of necrosis. The caecum was distended with serosal tears; the terminal ileum and caecum were resected. A side-to-side antimesenteric peristaltic anastomosis was performed. The patient remained clinically stable after surgery, with no post-operative complications and was discharged 11 days after initial presentation.

## Discussion

Heterotaxy syndrome, also known as situs ambiguous, can be classified into two subgroups: right isomerism and left isomerism.^3^ Right isomerism is characterised by isomerism of the right atrial appendage and is usually associated with asplenia. Left isomerism is characterised by isomerism of the left atrial appendage and is usually associated with polysplenia.^2^ Patients with heterotaxy syndrome have a wide range of cardiac and extra-cardiac manifestations.

The syndrome may be diagnosed antenatally.^2^ Right isomerism tends to present earlier with cyanosis or congestive cardiac failure due to complex congenital heart disease. Other presentations in infancy and childhood may be due to biliary atresia, volvulus or sepsis secondary to asplenia or hypo functioning polysplenia.^3^ Patients with left isomerism usually have less severe cardiac malformations and may present in adulthood with extra-cardiac complications.

Abnormal cardiac position is more common in right isomerism than left isomerism. Mesocardia or dextrocardia occur in 40% – 50% of patients with left isomerism.^2^ Azygous continuation of the IVC is commonly found in left isomerism, whereas bilateral superior vena cava (SVC) may occur in both left and right isomerism.^4^ Partial anomalous pulmonary venous connection can occur in left isomerism, while total anomalous pulmonary venous connection occurs commonly in right isomerism. Atrial septal defects (ASD) and atrioventricular septal defects (AVSD) are more common in right isomerism than left isomerism.^2^ The presented patient had levocardia, azygous continuation of the IVC, no anomalous pulmonary venous connections and no ASD or AVSD.

In right isomerism, bilateral trilobed lungs with bilateral right bronchus morphology may be found. In left isomerism, bilateral bilobed lungs with bilateral left bronchus morphology may be found.^2^ The presented case patient had a trilobed right lung and a bilobed left lung.

Asplenia is often seen in right isomerism and polysplenia in left isomerism. However, despite the quantity of splenic tissue present, it may be hypo or non-functioning. A centrally located liver is common in both right and left isomerism.^4^ The stomach may be left-sided, right-sided or central. The presented patient had multiple splenules, a left-sided liver, extending across the midline and a right-sided stomach.

Varying degrees of intestinal malrotation may be present in approximately 70% of patients with heterotaxy syndrome.^5^ According to Hill et al., the incidence of intestinal malrotation was found to be higher in right isomerism than left isomerism.^6^ Intestinal malrotation may predispose to midgut volvulus, although many patients may remain asymptomatic.

At our institution, patients with antenatal and early postnatal diagnosis of heterotaxy syndrome undergo routine screening for malrotation with an upper gastrointestinal (GI) contrast study in order to identify the position of the duodenojejunal junction. There has been much debate regarding the prophylactic Ladd’s procedure for patients with asymptomatic intestinal malrotation.^7^ The post-operative complication rate following elective Ladd’s procedure is high in patients with heterotaxy syndrome due to the concomitant cardiac anomalies and increased risk of sepsis related to reduced or absent splenic function.^8^

The nature of the cardiac malformations will determine the type of procedure required. Biventricular repair may be performed in patients with left isomerism with less severe cardiac malformations, whereas patients with right isomerism with more complex cardiac malformations often undergo a Fontan-type procedure.^1^

For patients with hypo or non-functioning spleens, various guidelines exist for prophylactic antimicrobial therapy during childhood and the requirement for additional vaccines in combination with the national immunisation programme schedule.^2^

The patient in this report presented for the first time in adulthood with an extra-cardiac complication which was bowel obstruction due to midgut volvulus. CT scan was the first imaging modality used to evaluate the patient in the emergency setting. The patient had some extra-cardiac features of left isomerism, that is, intestinal malrotation, a centrally located liver, a right-sided stomach and polysplenia. However, the patient had no radiologically appreciable cardiac abnormalities. The absence of cardiac malformations might be the reason for the late presentation in adulthood.

This case study demonstrates the diversity of clinical and radiological findings in patients with heterotaxy syndrome. Patients with heterotaxy syndrome do not always perfectly fit either of the classifications. Therefore, radiological evaluation is crucial to fully characterise the spectrum of abnormalities and plan the management approach. The incidence of heterotaxy syndrome may be underestimated and the number of diagnosed cases may increase due to increased utilisation of diagnostic imaging.

## Conclusion

Heterotaxy syndrome, a rare spectrum of congenital abnormalities, is important to be aware of in patients presenting with cardiac anomalies, intestinal malrotation and compromised immunity. Imaging plays an important role in fully evaluating the associated abnormalities, planning management and preventing complications in these patients.
